# Underactuated AUV Nonlinear Finite-Time Tracking Control Based on Command Filter and Disturbance Observer

**DOI:** 10.3390/s19224987

**Published:** 2019-11-15

**Authors:** Hao Xu, Guo-cheng Zhang, Jian Cao, Shuo Pang, Yu-shan Sun

**Affiliations:** Science and Technology on Underwater Vehicle Laboratory, Harbin Engineering University, Harbin 150001, China; xuhao0619@126.com (H.X.); sspp27@hotmail.com (S.P.); sunyushan@hrbeu.edu.cn (Y.-s.S.)

**Keywords:** underactuated AUV, 3D path following, backstepping method, super-twisting observer, ocean currents disturbance

## Abstract

The three-dimensional (3D) path following problem of an underactuated autonomous underwater vehicle with ocean currents disturbances is addressed in this paper. Firstly, the motion equation under the ocean currents disturbance is established, and the dynamic model of 3D tracking error is constructed based on virtual guidance method. Then, a finite-time control scheme based on super-twisting observer and command filtered backstepping technology is proposed. We adopt super-twisting observer based on finite-time theory to observe the ocean currents disturbances for improving the system robust. A command filtered backstepping is proposed to replace the differential process in the conventional backstepping method for avoiding the differential expansion problem. The filter compensation loop is designed to ensure the accuracy of the filtered signal, and the anti-integration saturation link is designed considering the influence of integral saturation. Lyapunov stability theory is used to prove the stability of the underactuated AUV. Simulation studies are conducted to show the effectiveness and robustness of the controller.

## 1. Introduction

Autonomous underwater vehicles have become more and more practical in many fields, such as civil and military, underwater monitoring, deep sea environmental resource exploration and development, ocean data observation and collection, seabed topographic scanning, submarine pipeline detection and marine mine clearance, etc. [1,2,3]. The completion of related tasks generally requires the AUV to follow an ideal path. Therefore, the path following technology of AUV has attracted the attention of many scholars and has become a hot research topic. AUVs are often designed to be under-actuated, such as the REMUS series [4,5], for reasons of energy savings, weight reduction and increased reliability. Most of underactuated AUVs cannot directly provide lateral and vertical forces, and can only control surge velocity, yaw angle velocity and pitch angle velocity directly. In addition, AUV is also affected by external disturbances such as waves, currents and uncertainties of its own hydrodynamic parameters in the actual environment, which increases the difficulty of controller design [6,7].

In the past few decades, researchers have tried various control methods for path following control of underactuated AUVs [8,9]. A cascade structure consisting of an integral LOS guidance and feedback linearized proportional derivative controller was designed in [9]. A tracking control law based on the extended state observer and the optimal reference signals were proposed in [10]. The control strategy based on the input-output feedback linearization method was designed in [11]. The robust adaptive path following method based on fuzzy position observer was proposed in [12]. The above path following controllers have achieved good control effects. However, most of them focus on two-dimensional path following problems such as horizontal or vertical planes. In the three-dimensional space, due to the strong nonlinearity and strong coupling characteristics of AUV, the path following control of under-actuated AUV is more challenging. The underactuated AUV nonlinear controller of 3D path following was proposed for the first time [13], but there were singularities. For solving the singularity problem, the literature [14] introduced virtual guidance points in the path and firstly proposed the line-of-sight (LOS) method. Subsequently, many researchers followed and improved the line-of-sight method in kinematic and introduced intelligent controller in dynamic. Improved line-of-sight (LOS) and fuzzy controller were designed in [15]. A 3D trajectory tracking controller for underactuated AUV was designed by linear stability theory and backstepping method [16]. However, the impacts of ocean currents were not considered in the above literatures. Designing an observer is one of the common means of nonlinear system control [17,18]. Sliding mode control is also one of the effective control methods [19]. For the ocean currents disturbance, the tracking control scheme combined with the sliding mode control and backstepping method was designed in [20], and adopted the fuzzy logic theory to estimate the nonlinear term and disturbance. In the literature [21], the tracking error equations of horizontal and vertical planes were established based on the line-of-sight method, the controller was designed by cascade theory and backstepping method. The literature [22] improved the LOS guidance law, introduced an integration strategy to eliminate the effects of currents, and designed a dynamic controller based on relative velocity. However, the above research can only achieve tracking of spatial lines and tracking control of curved paths cannot be realized. In addition, the process of calculating the derivative of a virtual control signal in the backstepping method is very complicated and the differential expansion problem may occur. The literature [23] present a command-filtering backstepping controller, by using numerical integration for the derivative of the virtual control signal, for avoiding the problem of differential expansion. But the integral saturation was not considered in this method. And the error between the filtered signal and the virtual control signal cannot guarantee to convergence.

At present, most controllers can only achieve asymptotic convergence, and cannot guarantee convergence in a limited time. The finite-time control method has gradually gained in-depth research in recent years because of its advantages of high accuracy, fast convergence speed and strong robustness [24]. The path following control with finite-time control theory was applied to the horizontal plane tracking, and achieve good control effects [25,26].

Motivated by the above discussions, the command filtering backstepping method, super-twisting observer and finite-time control technology were adopted to propose a three-dimensional path following controller for underactuated AUV in the presence of ocean currents, which has not been proposed in the published literatures. The main contributions of this article are:

(1) The differential filtering problem caused by the traditional backstepping calculation complexity is avoided by the command filtering backstepping method, and the part of filtering error compensation is proposed to ensure the accordance of virtual control signal and the filtered signal.
(2) Since the integral action of the filter part, the anti-integration saturation is considered in the control loop to deal with the problem of integral saturation in the control signals.
(3) A current disturbance observer is presented to reduce the impact of external ocean currents disturbances on the system and increase the tracking controller robustness.
(4) The 3D path following controller, based on the finite-time control theory, is designed to improve the response speed and control accuracy of the controller.

The rest of this paper is organized as follows. Firstly, the 5-DOF motion model and error model for AUV are established in Section 2. The main results, including the ocean currents disturbance observer and the 3D path following controller, are presented in Section 3. In Section 4, the mathematical proof of the control system is presented. Numerical simulation results, carried in MATLAB-Simulink to demonstrate the controller tracking performance, are given in Section 5. In the end, short conclusions are presented in Section 6.

## 2. Problem Formulation

### 2.1. Coordinate System and Parameter Definition

**Figure 1:**
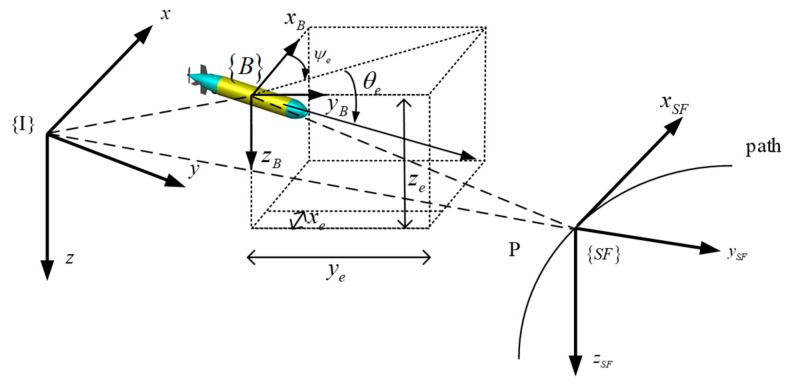
Three-dimensional (3D) path following frame of AUV.

As is depicted in Figure 1, {I} is assumed as the inertial reference frame (earth-fixed frame), its original point can be set at any place in the ocean. {B} is the body-fixed frame, its original point is set at the AUV’s gravity center. {SF} denotes the Serret-Frenet frame, its original point Q is the any point of the desired path. $\eta_{e}=\left(x,y,z\right)$ denotes the position of the AUV’s gravity center within the frame {I}. $\left(x_{P},y_{P},z_{P}\right)$ denotes the original center of {SF} within the inertial reference frame. $\left(x_{e},y_{e},z_{e}\right)$ denotes the position of the AUV’s gravity center within the Serret-Frenet frame {SF}.

### 2.2. AUV Kinematic and Dynamic Equations

According to Reference [27], the nonlinear motion equations of AUV can be described:

(1) $$\dot{\eta }=J(\eta )v,$$

(2) $$M\dot{v}+C(v)v+D(v)v+g(\eta )=\tau +\tau_{d}.$$

where η denotes the position and orientation vector with coordinates in the earth fixed frame, J(η) is the transform matrix between the body-fixed and inertial coordinates, v denotes the linear and angular velocity vector with coordinates in the body-fixed frame, M is the inertial and add inertial matrix, C(v) is the matrix od Coriolis and Centrifugal terms, D(v) is the matrix of hydrodynamics terms, g(η) is the vector of gravity and buoyant force, τ denotes the forces and moments in the body-fixed frame. It must be mentioned that the ocean currents were assumed constant in frame {B} in many earlier researches. A more natural and real assumption are made in this paper: the ocean currents are constant in frame {I}. The ocean currents in frame {I} are proposed as [27]

(3) $$v_{c}^{n}={\left[u_{c}^{n},v_{c}^{n},w_{c}^{n}\right]}^{T}.$$

With the Euler angle rotation matrix, it can be transformed to the frame {B}:

(4) $$\begin{array}{l} v_{c}^{b}=R_{b}^{n}(\psi ,\theta )\cdot v_{c}^{n}\\ \;=\left[\begin{array}{ccc} c\theta c\psi & -s\psi & s\theta c\psi \\ c\theta s\psi & c\psi & s\theta s\psi \\ -s\theta & 0 & c\theta \end{array}\right]\cdot {\left[u_{c}^{n},v_{c}^{n},w_{c}^{n}\right]}^{T} \end{array},$$

where $c^{*}$ and $s^{*}$ are abbreviations for $\cos^{*}$ and $\sin^{*}$, respectively. Assume the ocean currents change slowly, the acceleration of the current is negligible such that

(5) $${\dot{v}}_{c}^{b}\approx 0.$$

Usually, the roll motion of AUV can be ignored due to its left–right symmetrical structure [28]. Under the assumption, the motion equations can be given as

(6) $$\left\{ \begin{array}{l} \dot{x}=u_{r}c\theta c\psi -v_{r}s\psi +w_{r}s\theta c\psi \\ \dot{y}=u_{r}c\theta s\psi +v_{r}c\psi +w_{r}s\theta s\psi \\ \dot{z}=-u_{r}s\theta +w_{r}c\theta \\ \dot{\theta }=\dot{\alpha }+q\\ \dot{\psi }=\dot{\beta }+r/c\theta \end{array}\right.,$$

(7) $$\left\{ \begin{array}{l} \dot{u}=\frac{1}{m-X_{\dot{u}}}F_{u}+\frac{X_{uu}}{m-X_{\dot{u}}}u_{r}{}^{2}+\frac{X_{vv}}{m-X_{\dot{u}}}v_{r}{}^{2}+\frac{X_{ww}}{m-X_{\dot{u}}}w_{r}{}^{2}+\frac{X_{qq}}{m-X_{\dot{u}}}q^{2}\\ \dot{v}=-\frac{m-Y_{ur}}{m-Y_{\ddot{v}}}u_{r}r+\frac{Y_{uv}}{m-Y_{\ddot{v}}}u_{r}v+\frac{Y_{v\left|v\right|}v_{r}}{m-Y_{\ddot{v}}}\left|v_{r}\right|\\ \dot{w}=-\frac{m-Z_{uq}}{m-Z_{\dot{w}}}u_{r}q+\frac{Z_{uw}}{m-Z_{\dot{w}}}u_{r}w_{r}+\frac{Z_{w\left|w\right|}}{m-Z_{\dot{w}}}w_{r}\left|w_{r}\right|+\frac{1}{m-Z_{\dot{w}}}mz_{g}q^{2}\\ \dot{q}=\frac{1}{I_{y}-M_{\dot{q}}}M+\frac{M_{q\left|q\right|}}{I_{y}-M_{\dot{q}}}q\left|q\right|-\frac{M_{uq}}{I_{y}-M_{\dot{q}}}u_{r}q-\frac{M_{uw}}{I_{y}-M_{\dot{q}}}u_{r}w_{r}-\\ \;\frac{1}{I_{y}-M_{\dot{q}}}(z_{g}w-z_{b}B)s\theta -\frac{1}{I_{y}-M_{\dot{q}}}mz_{g}(w_{r}q-v_{r}r)\\ \dot{r}=\frac{1}{I_{z}-N_{\dot{r}}}N+\frac{N_{uv}}{I_{z}-N_{\dot{r}}}u_{r}v_{r}+\frac{N_{v\left|v\right|}}{I_{z}-N_{\dot{r}}}v_{r}\left|v_{r}\right|+\frac{N_{ur}}{I_{z}-N_{\dot{r}}}u_{r}r \end{array}\right.,$$

where m is the AUV mass, $I_{y}$ and $I_{z}$ denote the inertia moments about the pitch and yaw rotation. $u_{r}$, $v_{r}$ and $w_{r}$ denote the real linear velocities, where $u_{r}=u-u_{c}^{b}$, $v_{r}=v-v_{c}^{b}$ and $w_{r}=w-w_{c}^{b}$. q, r are the velocities of pitch and yaw. r and ψ present the pitch and yaw angle. The corresponding hydrodynamic derivatives are denoted by $X_{\left(*\right)}$, $Y_{\left(*\right)}$, $Z_{\left(*\right)}$, $M_{\left(*\right)}$ and $N_{\left(*\right)}$. $z_{g}$ and $z_{b}$ are the vertical positions of the gravity and buoyancy center within body-fixed frame. X denotes the force generated by the stern thruster. M and N are the moments provided by horizontal and vertical rudders.

### 2.3. AUV Error Systems

The error formulations can be derived by using the Serret-Frenet frame {SF} according to Figure 1 [29]. We define point P that is the origin of {SF} as the virtual moving target AUV that describes the path, and the position in {I} is $\eta_{d}^{e}={\left(x_{d},y_{d},z_{d}\right)}^{T}$. The Serret-Frenet frame is rotated with angle $\theta_{F}$, $\psi_{F}$ relative to the inertial frame.

(8) $$\left\{ \begin{array}{l} \theta_{F}=-\arctan\left(\frac{{\dot{z}}_{d}}{\sqrt{{\dot{x}}_{d}^{2}+{\dot{y}}_{d}^{2}}}\right)\\ \psi_{F}=\arctan\left(\frac{{\dot{y}}_{d}}{{\dot{x}}_{d}}\right) \end{array}\right..$$

The desired path is described by parameter s. Rotation angles can be also defined as

(9) $$\left\{ \begin{array}{l} {\dot{\theta }}_{F}=c_{1}(s)\dot{s}\\ {\dot{\psi }}_{F}=c_{2}(s)\dot{s} \end{array}\right.,$$

where $c_{1}(s)$ and $c_{2}(s)$ are the torsion and curvature of the virtual target point in spatial curve.

The AUV’s position is $\eta^{e}={\left(x,y,z\right)}^{T}$, and represented by point Q. The position of the AUV is usually obtained by GPS, but GPS is not available when the AUV is underwater. At that time, it can only be obtained by dead reckoning or long/short baseline instruments [30]. Hence, the error of path following can be defined in frame {B}.

(10) $$\varepsilon =R_{b}^{eT}(\eta^{e}-\eta_{d}^{e}),$$

(11) $$\dot{\varepsilon }={\dot{R}}_{b}^{eT}(\eta^{e}-\eta_{d}^{e})+R_{b}^{eT}({\dot{\eta }}^{e}-{\dot{\eta }}_{d}^{e}),$$

where $R_{b}^{e}$ is the rotation matrix from {B} to {I}. ${\dot{R}}_{b}^{e}=R_{b}^{e}{\left[\varpi \right]}_{qr}$, ${\left[\varpi \right]}_{qr}$ is the vector of angular velocity. ${\dot{\eta }}^{e}=R_{b}^{e}v_{b}$, $v_{b}={\left(u,v,w\right)}^{T}$ is the vector of velocities in {B}. ${\dot{\eta }}_{d}^{e}=R_{F}^{e}v_{F}$, $v_{F}={\left(u_{r},0,0\right)}^{T}$ is the reference velocities. $R_{F}^{e}$ is the rotation matrix from {SF} to {I}.

(12) $$\begin{array}{l} \dot{\tau }={\left[\varpi \right]}_{qr}^{T}R_{b}^{eT}(\eta^{e}-\eta_{d}^{e})+R_{b}^{eT}({\dot{\eta }}^{e}-{\dot{\eta }}_{d}^{e})\\ \;={\left[\varpi \right]}_{qr}^{T}\tau +R_{b}^{eT}R_{b}^{e}v_{b}-R_{b}^{eT}R_{F}^{e}v_{F}\\ \;={\left[\varpi \right]}_{qr}^{T}\tau +v_{b}-R(\psi_{e},\theta_{e})v_{F} \end{array},$$

where ${\left[\varpi \right]}_{qr}=\left[\begin{array}{ccc} 0 & r & -q\\ -r & 0 & 0\\ q & 0 & 0 \end{array}\right]$, $R_{b}^{eT}R_{F}^{e}=R(\psi_{e},\theta_{e})$, $\psi_{e}=-\psi_{F}+\psi +\beta$ and $\theta_{e}=\theta +\alpha -\theta_{F}$. $R(\psi_{e},\theta_{e})=\left(\begin{array}{ccc} c\theta_{e}c\psi_{e} & -s\psi_{e} & s\theta_{e}c\psi_{e}\\ c\theta_{e}s\psi_{e} & c\psi_{e} & s\theta_{e}s\psi_{e}\\ -s\theta_{e} & 0 & c\theta_{e} \end{array}\right)$.

(13) $$\left[\begin{array}{c} {\dot{x}}_{e}\\ {\dot{y}}_{e}\\ {\dot{z}}_{e} \end{array}\right]=\left[\begin{array}{ccc} 0 & r & -q\\ -r & 0 & 0\\ q & 0 & 0 \end{array}\right]\left[\begin{array}{c} x_{e}\\ y_{e}\\ z_{e} \end{array}\right]+\left[\begin{array}{c} u\\ v\\ w \end{array}\right]-R(\psi_{e},\theta_{e})\left[\begin{array}{c} u_{r}\\ 0\\ 0 \end{array}\right].$$

The angular velocities of virtual target are presented as Equation (9). Then we define the AUV path following error:

(14) $$\left\{ \begin{array}{l} {\dot{x}}_{e}=ry_{e}-qz_{e}+u-u_{r}c\psi_{e}c\theta_{e}\\ {\dot{y}}_{e}=-rx_{e}+v-u_{r}s\psi_{e}c\theta_{e}\\ {\dot{z}}_{e}=qx_{e}+w+u_{r}s\theta_{e}\\ {\dot{\psi }}_{e}=-c_{1}(s)\dot{s}+r/c\theta_{e}+\dot{\beta }\\ {\dot{\theta }}_{e}=-c_{2}(s)\dot{s}+q+\dot{\alpha } \end{array}\right.,$$

**Lemma** **1.** *For nonlinear systems*$\dot{\omega }=g(\omega ,\tau )$*, where*ω*denotes the state vector, and*τ*is the control input,*g(·)*is continuous and*g(0)=0*, if*λ>0*,*0<α<1*and*0<η<∞*, and the continuous function*V(ω)*meets*$\dot{V}(\omega )\leq -\lambda V^{\alpha }(\omega )+\eta$*, then the system converges in finite-time [31]*.

**Lemma** **2.** *For the system in lemma 1, if*$\lambda_{1}>0$*,*$\lambda_{2}>0$*and*0<α<1*, and the continuous function*V(ω)*meets*$\dot{V}(\omega )\leq -\lambda_{1}V(\omega )-\lambda_{2}V^{\alpha }(\omega )$*, then the system converges in finite-time. The time of convergence is*$T\leq \frac{1}{\lambda_{1}(1-\alpha )}\ln\frac{\left(\lambda_{1}V^{1-\alpha }(\omega_{0})+\lambda_{2}\right)}{\lambda_{2}}$*, where the*$V(\omega_{0})$*is the initial value of V(ω) [32]*.

## 3. Design of Path Following Control 

**Figure 2:**
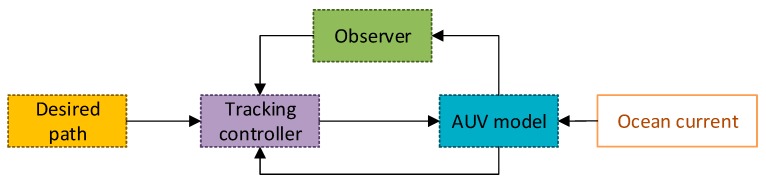
The control flow chart.

This section introduces the nonlinear control law to solve the path following control problem of underactuated AUV in the presence of the ocean current. The control flow chart is shown in Figure 2. Controller design is divided into two stages. The first stage handles the design of the ocean current observer based on super-twisting technology. Observer is used to compensate for the effects of currents on the system. The second stage addresses the tracking controller based on command filtered backstepping. Taking the designed virtual control as the input of the command filter, the derivative of the virtual control is obtained through the integration rather than the differential process. 

Controller of the AUV is developed in this section such that the real AUV can track the virtual AUV, namely

(15) $$\underset{t\in \left[t_{0},\infty \right]}{\sup}(\left\|x_{e}\right\|,\left\|y_{e}\right\|,\left\|z_{e}\right\|,\left\|u-u_{d}\right\|)\leq (\varepsilon_{1},\varepsilon_{2},\varepsilon_{3},\varepsilon_{4}),$$

where $\varepsilon_{i}(i=1,2,3,4)$ denote arbitrary small positive numbers.

Referring to the LOS guidance theory, pitch and heading guidance laws are defined as

(16) $$\left\{ \begin{array}{l} \theta_{LOS}=-\theta_{a}\frac{e^{2k_{\theta }z_{e}}-1}{e^{2k_{\theta }z_{e}}+1}\\ \psi_{LOS}=-\psi_{a}\frac{e^{2k_{\psi }y_{e}}-1}{e^{2k_{\psi }y_{e}}+1} \end{array}\right.,$$

where $\theta_{a}\in \left(0,\frac{\pi }{2}\right)$ and $\psi_{a}\in \left(0,\frac{\pi }{2}\right)$ are chosen to let $\theta_{LOS}\in \left(-\frac{\pi }{2},\frac{\pi }{2}\right)$ and $\psi_{LOS}\in \left(-\frac{\pi }{2},\frac{\pi }{2}\right)$, $k_{\theta }>0$ and $k_{\psi }>0$ are gains. When the vertical error between AUV and desired path becomes larger, $\theta_{LOS}$ also increases, and when AUV is in the desired path, the error becomes zero, and $\theta_{LOS}=0$. $\psi_{LOS}$ is the same. AUV will have better endurance and the redundant range can be shortened to save energy by introducing the approach angle [29].

**Figure 3:**
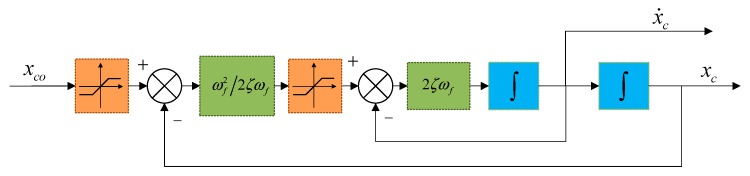
The command filter structure.

In an attempt to eliminate effects of differential expansion in the conventional method, a command filter is considered to add to the backstepping control loop. In this approach, the control signals are passed via filter to get derivative instead of the differentiation process.

(17) $$\left\{ \begin{array}{l} {\dot{z}}_{1}=z_{2}\\ {\dot{z}}_{2}=-2\zeta \omega_{f}z_{2}-\omega_{f}^{2}(z_{1}-x_{co}) \end{array}\right.$$

where $x_{co}={\left[\begin{array}{cccc} \begin{array}{ccc} \begin{array}{ccc} x_{ce}^{o} & y_{ce}^{o} & z_{ce}^{o} \end{array} & \psi_{ce}^{o} & \theta_{ce}^{o} \end{array} & u_{c}^{o} & r_{c}^{o} & q_{c}^{o} \end{array}\right]}^{T}$ is the vector of desired control, $z_{1}=x_{c}={\left[\begin{array}{cccc} \begin{array}{ccc} \begin{array}{ccc} x_{ce} & y_{ce} & z_{ce} \end{array} & \psi_{ce} & \theta_{ce} \end{array} & u_{c} & r_{c} & q_{c} \end{array}\right]}^{T}$ and $z_{2}={\dot{x}}_{c}={\left[\begin{array}{cccc} \begin{array}{ccc} \begin{array}{ccc} {\dot{x}}_{ce} & {\dot{y}}_{ce} & {\dot{z}}_{ce} \end{array} & {\dot{\psi }}_{ce} & {\dot{\theta }}_{ce} \end{array} & {\dot{u}}_{c} & {\dot{r}}_{c} & {\dot{q}}_{c} \end{array}\right]}^{T}$ are the filtered control signals vector. ζ and *ω_f_* are the filter parameters, where 0 < *ζ* < 1, *ω_f_* > 0. The flow chart of command filter is shown as Figure 3.

### 3.1. Design of Super-Twisting Disturbance Observer

In this section, an ocean currents observer based on super-twisting technology is designed. Let $\hat{D}$ and $\hat{x}$ denote the observation of D and x, we can get

(18) $$\dot{\hat{x}}=A\hat{x}+Bu+C+\hat{D},$$

where, $A=-M^{-1}(D(v)+C(v))$, $B=M^{-1}$, $C=-M^{-1}g(\eta )$, $\hat{D}=M^{-1}{\hat{\tau }}_{d}$. Error vector of state x and observation error $\tilde{D}$ can be present as

(19) $$\begin{array}{l} e=x-\hat{x}\\ \tilde{D}=D-\hat{D} \end{array}.$$

The observer of disturbance was designed as

(20) $${\hat{D}}_{i}=k_{1}\sqrt{\left|e_{i}\right|}\mathrm{sgn}(e_{i})+\int k_{2}\mathrm{sgn}(e_{i})dt,i=1,2,3,$$

where $k_{1}$ and $k_{2}$ are positive parameters. 

Together with Equations (18) and (19), we have

(21) $${\dot{e}}_{i}={\dot{x}}_{i}-{\dot{\hat{x}}}_{i}=D_{i}-k_{i}\sqrt{\left|e_{i}\right|}\mathrm{sgn}(e_{i})-\int k_{2}\mathrm{sgn}(e_{i})dt,i=1,2,3.$$

Obviously, when the observation error of x is finite-time converged, the observation error of disturbance also converges in finite-time. Following is the proof.

**Proof.**  For convenience of proof, select the intermediate quantities $M\in R^{3\times 1}$ and $N\in R^{3\times 1}$

(22) $$\begin{array}{l} M_{i}=k_{1}\sqrt{\left|e_{i}\right|}\mathrm{sgn}(e_{i})\\ N_{i}=D_{i}-\frac{1}{2}\int k_{2}\mathrm{sgn}(e_{i}) \end{array}.$$

Differentiating the above equation, we can get

(23) $$\begin{array}{l} {\dot{M}}_{i}=\frac{1}{2\left|M_{i}\right|}{\dot{e}}_{i}=\frac{1}{2\left|M_{i}\right|}(-k_{1}M_{i}+N_{i})\\ {\dot{N}}_{i}={\dot{D}}_{i}-\frac{1}{2}k_{2}\mathrm{sgn}(e_{i}) \end{array}.$$

Let $\beta_{i}={\left[M_{i}N_{i}\right]}^{T}$, and differentiate it:

(24) $$\begin{array}{l} {\dot{\beta }}_{i}=\frac{1}{2\left|M_{i}\right|}\left[\begin{array}{cc} -k_{1} & 1\\ -k_{2} & 0 \end{array}\right]\left[\begin{array}{c} M_{i}\\ N_{i} \end{array}\right]+\\ \frac{1}{2\left|M_{i}\right|}\left[\begin{array}{cc} 0 & 0\\ 0 & 2\left|M_{i}\right| \end{array}\right]\left[\begin{array}{c} 0\\ {\dot{D}}_{i} \end{array}\right] \end{array}.$$

$D_{i}$ is bounded, $\left|{\dot{D}}_{i}\right|<\Delta$, let 0<ρ<2Δ. Hence, ${\dot{D}}_{i}=\frac{\rho }{2}\mathrm{sgn}(e_{i})=\frac{\rho }{2}\frac{M_{i}}{\left|M_{i}\right|}$, we choose $A=\frac{1}{2\left|M_{i}\right|}\left[\begin{array}{cc} -k_{1} & 1\\ -k_{2}+\rho & 0 \end{array}\right]$

(25) $${\dot{\beta }}_{i}=A\beta_{i}.$$

Consider the Lyapunov function as follows

(26) $$V_{i0}=\beta_{i}^{T}P\beta_{i},$$

where $P=\left[\begin{array}{cc} \lambda +\tau^{2} & -\tau \\ -\tau & 1 \end{array}\right]$ is the symmetric matrix.

Differentiating $V_{i0}$ yields

(27) $${\dot{V}}_{i0}=\beta_{i}^{T}\left[A^{T}P+PA\right]\beta_{i}.$$

Choose $Q=\left[\begin{array}{cc} 2k_{1}\lambda +2\tau \rho & -\rho -\lambda -\tau^{2}\\ -\rho -\lambda -\tau^{2} & 2\tau \end{array}\right]$, when $k_{1}>\frac{\left(\rho +\lambda +\tau^{2}\right)}{2\lambda \tau }+\frac{\tau }{2\lambda }-\frac{\tau \rho }{\lambda }$ and $\lambda_{\min}(Q)\geq \tau$, we get

(28) $${\dot{V}}_{i0}=\beta_{i}^{T}\left[A^{T}P+PA\right]\beta_{i}\leq -\frac{1}{2\left|M_{i}\right|}\beta_{i}^{T}Q\beta_{i}\leq -\frac{\tau }{2\left|M_{i}\right|}{\left|\beta_{i}\right|}^{2}.$$

Equation (26) meets the following conditions

(29) $$\lambda_{\min}(P){\left\|\beta_{i}\right\|}^{2}\leq \beta_{i}^{T}P\beta_{i}\leq \lambda_{\max}(P){\left\|\beta_{i}\right\|}^{2}.$$

Then

(30) $$\left\{ \begin{array}{l} \left|M_{i}\right|\leq \left\|\beta_{i}\right\|\\ \frac{V_{i0}^{1/2}}{\lambda_{\max}^{1/2}}\leq \left\|\beta_{i}\right\| \end{array}\right..$$

According to Equations (28) and (30), we get

(31) $$\left\{ \begin{array}{l} {\dot{V}}_{i0}\leq -\mu V_{i0}^{1/2}\\ \mu =\frac{\tau }{2\lambda_{\max}^{1/2}(P)} \end{array}\right.,$$

where μ>0, at this moment, Equation (31) meets the lemma 1. Hence, the error of system states and observation can converge in finite-time. The disturbance error converges in a small neighborhood, and defined as $\left|\tilde{D}\right|\leq \zeta_{M}$. □

### 3.2. Position Control

According to Equation (14), we design the Lyapunov function:

(32) $$V_{p}=\frac{1}{2}(x_{e}^{2}+y_{e}^{2}+z_{e}^{2})$$

Then, differentiate $V_{p}$ along with Equation (14), we can get:

(33) $${\dot{V}}_{p}=x_{e}(u-u_{r}c\psi_{e}c\theta_{e})+y_{e}(-u_{r}s\psi_{e}c\theta_{e}+v)+z_{e}(u_{r}s\theta_{e}+w)$$

According to References [8], the virtual control signals are designed as:

(34) $$\left\{ \begin{array}{l} \psi_{ce}^{o}=\arcsin\frac{k_{v2}z_{e}}{\sqrt{1+{\left(k_{v2}z_{e}\right)}^{2}}}\\ \theta_{ce}^{o}=-\arcsin\frac{k_{v3}y_{e}}{\sqrt{1+{\left(k_{v3}y_{e}\right)}^{2}}}\\ u_{c}^{o}=-k_{v1}+u_{r}c\psi_{ce}^{o}c\theta_{ce}^{o} \end{array}\right.$$

where $k_{v1}>0$, $k_{v2}>0$ and $k_{v3}>0$ are constant gains, and substitute Equation (34) into Equation (33), we get

(35) $$\begin{array}{l} {\dot{V}}_{p}=-k_{v1}x_{e}^{2}-k_{v2}u_{r}\frac{1}{\sqrt{1+(k_{v2}y_{e}^{2})}}\frac{1}{\sqrt{1+(k_{v3}z_{e}^{2})}}y_{e}^{2}-\\ \;k_{3}u_{r}\frac{1}{\sqrt{1+(k_{v3}z_{e}^{2})}}+y_{e}v+z_{e}w \end{array}$$

For avoiding the differential expansion problem, command filtered backstepping method is used to replace the differential process in the conventional backstepping method.

We define the tracking error of position:

(36) $$\left[\begin{array}{c} {\tilde{x}}_{e}\\ {\tilde{y}}_{e}\\ {\tilde{z}}_{e} \end{array}\right]=\left[\begin{array}{c} x_{e}-x_{ce}\\ y_{e}-y_{ce}\\ z_{e}-z_{ce} \end{array}\right].$$

Then, differentiating the above equation alone with Equation (14) yields following error systems.

(37) $$\begin{array}{l} \left[\begin{array}{c} {\dot{\tilde{x}}}_{e}\\ {\dot{\tilde{y}}}_{e}\\ {\dot{\tilde{z}}}_{e} \end{array}\right]=\left[\begin{array}{c} r{\tilde{y}}_{e}-q{\tilde{z}}_{e}\\ -r{\tilde{x}}_{e}\\ q{\tilde{x}}_{e} \end{array}\right]+\\ \left[\begin{array}{ccc} A & Bg(\tilde{\psi })u_{r} & Cg(\tilde{\theta })u_{r} \end{array}\right]\left[\begin{array}{c} \tilde{u}\\ \tilde{\psi }\\ \tilde{\theta } \end{array}\right] \end{array},$$

where $\tilde{u}=u-u_{ce}$, $\tilde{\psi }=\psi_{e}-\psi_{ce}$, $\tilde{\theta }=\theta_{e}-\theta_{ce}$ are the errors of filter, and

$$\begin{array}{l} A=\left[\begin{array}{c} 1\\ 0\\ 0 \end{array}\right],B=\left[\begin{array}{cc} \begin{array}{c} c\psi_{ce}c\theta_{e}\\ s\psi_{ce}c\theta_{e}\\ 0 \end{array} & \begin{array}{c} -s\psi_{ce}c\theta_{e}\\ c\psi_{ce}c\theta_{e}\\ 0 \end{array} \end{array}\right],C=\left[\begin{array}{cc} \begin{array}{c} c\theta_{ce}c\psi_{ce}\\ c\theta_{ce}s\psi_{e}\\ -s\theta_{ce} \end{array} & \begin{array}{c} -s\theta_{ce}c\psi_{ce}\\ -s\theta_{ce}s\psi_{e}\\ -c\theta_{ce} \end{array} \end{array}\right]\\ g(\tilde{\psi })={\left[\begin{array}{cc} \frac{c\tilde{\psi }-1}{\tilde{\psi }} & \frac{s\tilde{\psi }}{\tilde{\psi }} \end{array}\right]}^{T},g(\tilde{\theta })={\left[\begin{array}{cc} \frac{c\tilde{\theta }-1}{\tilde{\theta }} & \frac{s\tilde{\theta }}{\tilde{\theta }} \end{array}\right]}^{T} \end{array},$$

where $\underset{\tilde{\psi }\to 0}{\lim}g(\tilde{\psi })={\left[\begin{array}{cc} 0 & 1 \end{array}\right]}^{T}$, $\underset{\tilde{\psi }\to 0}{\lim}g(\tilde{\theta })={\left[\begin{array}{cc} 0 & 1 \end{array}\right]}^{T}$.

Then, by differentiating Equation (36) and using ${\left[\begin{array}{ccc} {\dot{x}}_{ce}^{o} & {\dot{y}}_{ce}^{o} & {\dot{z}}_{ce}^{o} \end{array}\right]}^{T}$, the following equations are obtained.

(38) $$\left[\begin{array}{c} {\dot{x}}_{e}\\ {\dot{y}}_{e}\\ {\dot{z}}_{e} \end{array}\right]=\left[\begin{array}{c} {\dot{\tilde{x}}}_{e}\\ {\dot{\tilde{y}}}_{e}\\ {\dot{\tilde{z}}}_{e} \end{array}\right]+\left[\begin{array}{c} {\dot{x}}_{ec}-{\dot{x}}_{ce}^{o}\\ {\dot{y}}_{ec}-{\dot{y}}_{ce}^{o}\\ {\dot{z}}_{ec}-{\dot{z}}_{ce}^{o} \end{array}\right]+\left[\begin{array}{c} {\dot{x}}_{ce}^{o}\\ {\dot{y}}_{ce}^{o}\\ {\dot{z}}_{ce}^{o} \end{array}\right].$$

The desired signal ${\left[\begin{array}{ccc} {\dot{x}}_{ce}^{o} & {\dot{y}}_{ce}^{o} & {\dot{z}}_{ce}^{o} \end{array}\right]}^{T}$ can be written as:

(39) $$\left[\begin{array}{c} {\dot{x}}_{ce}^{o}\\ {\dot{y}}_{ce}^{o}\\ {\dot{z}}_{ce}^{o} \end{array}\right]=\left[\begin{array}{c} -k_{x}{\tilde{x}}_{e}+{\dot{x}}_{ce}\\ -k_{y}{\tilde{y}}_{e}+{\dot{y}}_{ce}\\ -k_{z}{\tilde{z}}_{e}+{\dot{z}}_{ce} \end{array}\right].$$

Substitute Equations (37) and (39) into Equation (38), and the errors of position are yielded as

(40) $$\begin{array}{c} \left[\begin{array}{c} {\dot{\tilde{x}}}_{e}\\ {\dot{\tilde{y}}}_{e}\\ {\dot{\tilde{z}}}_{e} \end{array}\right]=\left[\begin{array}{c} r{\tilde{y}}_{e}-q{\tilde{z}}_{e}\\ -r{\tilde{x}}_{e}\\ q{\tilde{x}}_{e} \end{array}\right]+\left[\begin{array}{c} -k_{x}{\tilde{x}}_{e}\\ -k_{y}{\tilde{y}}_{e}\\ -k_{z}{\tilde{z}}_{e} \end{array}\right]+\left[\begin{array}{c} {\dot{x}}_{ce}-{\dot{x}}_{ce}^{o}\\ {\dot{y}}_{ce}-{\dot{y}}_{ce}^{o}\\ {\dot{z}}_{ce}-{\dot{z}}_{ce}^{o} \end{array}\right]+\\ \left[\begin{array}{ccc} A & Bg(\tilde{\psi })u_{r} & Cg(\tilde{\theta })u_{r} \end{array}\right]\left[\begin{array}{c} \tilde{u}\\ \tilde{\psi }\\ \tilde{\theta } \end{array}\right] \end{array}.$$

### 3.3. Attitude Control

Differentiate $\tilde{\psi }=\psi_{e}-\psi_{ce}$ and $\tilde{\theta }=\theta_{e}-\theta_{ce}$, we get

(41) $$\dot{\tilde{\psi }}=(r_{c}^{o}+(r_{c}-r_{c}^{o})+\tilde{r})/c\theta +\dot{\beta }-r_{F}-{\dot{\psi }}_{ce},$$

(42) $$\dot{\tilde{\theta }}=q_{c}^{o}+(q_{c}-q_{c}^{o})+\tilde{q}-q_{F}-{\dot{\theta }}_{ce},$$

where the errors of angular velocities are defined as $\tilde{r}=r-r_{c}$, $\tilde{q}=q-q_{c}$.

Based on Equations (41) and (42), $q_{c}^{o}$ and $r_{c}^{o}$ represent the desired virtual control signals of angular velocities q and r, and can be proposed as

(43) $$\begin{array}{l} r_{c}^{o}=c\theta (r_{F}+{\dot{\psi }}_{c}+\dot{\beta }-k_{\psi }\tilde{\psi })-\psi_{bs}\\ q_{c}^{o}=q_{F}+{\dot{\theta }}_{c}-k_{\theta }\tilde{\theta }-\theta_{bs} \end{array},$$

where $k_{\psi }>0$, $k_{\theta }>0$ are the controller parameters, $\psi_{bs}$ and $\theta_{bs}$ represent the robust terms. The robust terms are defined in Section 4.

Substitute Equation (43) into Equations (41) and (42), Equations (41) and (42) can be rewritten as

(44) $$\begin{array}{l} \dot{\tilde{\psi }}=-k_{\psi }\tilde{\psi }+\frac{(r_{c}-r_{c}^{o})+\tilde{r}}{c\theta }-\psi_{bs}\\ \dot{\tilde{\theta }}=-k_{\theta }\tilde{\theta }+(q_{c}-q_{c}^{o})+\tilde{q}-\theta_{bs} \end{array}.$$

### 3.4. Velocity and Angular Velocity Control

For improving the robustness of the controller, we design the integral term $\varepsilon_{1}$, $\varepsilon_{2}$ and $\varepsilon_{3}$, where ${\dot{\varepsilon }}_{1}=\tilde{u}$, ${\dot{\varepsilon }}_{2}=\tilde{q}$, ${\dot{\varepsilon }}_{3}=\tilde{r}$. The finite-time control input of AUV 3D path following can be proposed as

(45) $$\left\{ \begin{array}{l} F_{u}=(m-X_{\dot{u}})(-k_{u}\tilde{u}-k_{fu}{\mathrm{sig}}^{\gamma }(\tilde{u})+{\dot{u}}_{c}-u_{bs})-f_{u}\\ M=(I_{y}-M_{\dot{q}})(-k_{q}\tilde{q}-k_{fq}{\mathrm{sig}}^{\gamma }(\tilde{q})+{\dot{q}}_{c}-q_{bs})-f_{q}\\ N=(I_{z}-N_{\dot{r}})(-k_{r}\tilde{r}-k_{fr}{\mathrm{sig}}^{\gamma }(\tilde{r})+{\dot{r}}_{c}-r_{bs})-f_{r} \end{array}\right.,$$

where $f_{u}=X_{uu}u_{r}{}^{2}+X_{vv}v_{r}{}^{2}+X_{ww}w_{r}{}^{2}+X_{qq}q^{2}$, $f_{q}=-(z_{g}w-z_{b}B)\sin\theta +M_{q\left|q\right|}q\left|q\right|-M_{uw}u_{r}w_{r}-M_{uq}u_{r}q-mz_{g}(w_{r}q-v_{r}r)$ and $f_{r}=N_{uv}u_{r}v_{r}+N_{v\left|v\right|}v_{r}\left|v_{r}\right|+N_{ur}u_{r}r$ are nonlinear dynamic terms. ${\mathrm{sig}}^{\gamma }(x)$ is given as

(46) $${\mathrm{sig}}^{\gamma }(x)=\mathrm{sgn}(x){\left|x\right|}^{\gamma }.$$

where x∈ℝ, α∈(0,1), and ${\mathrm{sig}}^{\alpha }(\cdot )$ is continuous and increasing, and ${\mathrm{sig}}^{\alpha }(0)=0$.

Considering the observer into the Equation (45), the controller of the underactuated AUV proposed in this paper can be obtained:

(47) $$\left\{ \begin{array}{l} F_{u}=(m-X_{\dot{u}})(-k_{u}\tilde{u}-k_{fu}{\mathrm{sig}}^{\gamma }(\tilde{u})+{\dot{u}}_{c}-u_{bs}-{\hat{F}}_{u})-f_{u}\\ M=(I_{y}-M_{\dot{q}})(-k_{q}\tilde{q}-k_{fq}{\mathrm{sig}}^{\gamma }(\tilde{q})+{\dot{q}}_{c}-q_{bs}-\hat{N})-f_{q}\\ N=(I_{z}-N_{\dot{r}})(-k_{r}\tilde{r}-k_{fr}{\mathrm{sig}}^{\gamma }(\tilde{q})+{\dot{r}}_{c}-r_{bs}-\hat{M})-f_{r} \end{array}\right.,$$

When the command filter controller is not considered, Equation (47) is changed as follows

(48) $$\left\{ \begin{array}{l} F_{u}=(m-X_{\dot{u}})(-k_{u}\tilde{u}-k_{fu}{\mathrm{sig}}^{\gamma }(\tilde{u})+{\dot{u}}_{c}-{\hat{F}}_{u})-f_{u}\\ M=(I_{y}-M_{\dot{q}})(-k_{q}\tilde{q}-k_{fq}{\mathrm{sig}}^{\gamma }(\tilde{q})+{\dot{q}}_{c}-\hat{N})-f_{q}\\ N=(I_{z}-N_{\dot{r}})(-k_{r}\tilde{r}-k_{fr}{\mathrm{sig}}^{\gamma }(\tilde{q})+{\dot{r}}_{c}-\hat{M})-f_{r} \end{array}\right.,$$

Then we substitute Equation (45) into Equation (7) and get the following error dynamics

(49) $$\left\{ \begin{array}{l} \dot{\tilde{u}}=-k_{u}\tilde{u}-k_{fu}{\mathrm{sig}}^{\gamma }(\tilde{u})-u_{bs}\\ \dot{\tilde{q}}=-k_{q}\tilde{q}-k_{fq}{\mathrm{sig}}^{\gamma }(\tilde{q})-q_{bs}\\ \dot{\tilde{r}}=-k_{r}\tilde{r}-k_{fr}{\mathrm{sig}}^{\gamma }(\tilde{r})-r_{bs} \end{array}\right..$$

Let $\sigma_{u}=\tilde{u}$, $\sigma_{r}=\tilde{r}$ and $\sigma_{q}=\tilde{q}$, we get the filter error compensation dynamics

(50) $$\left\{ \begin{array}{l} {\dot{\sigma }}_{u}=-k_{u}\sigma_{u}-k_{fu}{\mathrm{sig}}^{\gamma }(\sigma_{u})-u_{bs}\\ {\dot{\sigma }}_{q}=-k_{q}\sigma_{q}-k_{fq}{\mathrm{sig}}^{\gamma }(\sigma_{q})-q_{bs}\\ {\dot{\sigma }}_{r}=-k_{r}\sigma_{r}-k_{fr}{\mathrm{sig}}^{\gamma }(\sigma_{r})-r_{bs} \end{array}\right..$$

The above Equation (50) can be finally rewritten as

(51) $$\dot{E}=GE+HT+Jsig^{\gamma }(E),$$

where $G=-K_{P}=diag\{-k_{u},-k_{q},-k_{r}\}$, $k_{u}$, $k_{q}$ and $k_{r}$ are positive parameters. $H=I_{3\times 3}$, $J=-K_{F}=diag\{-k_{fu},-k_{fq},-k_{fr}\}$, $k_{fu}$, $k_{fq}$ and $k_{fr}$ are positive parameters. $T={\left[-u_{bs},-q_{bs},-r_{bs}\right]}^{T}$, $E={\left[\sigma_{u},\sigma_{q},\sigma_{r}\right]}^{T}$.

### 3.5. Design of Compensation Loop

For guaranteeing that the error between the desired signals and the filter signals can be converged, a compensation of filter error is proposed in the part. The errors of compensation are defined as following

(52) $$\left[\begin{array}{c} \sigma_{x}\\ \sigma_{y}\\ \sigma_{z} \end{array}\right]=\left[\begin{array}{c} {\tilde{x}}_{e}-\zeta_{x}\\ {\tilde{y}}_{e}-\zeta_{y}\\ {\tilde{z}}_{e}-\zeta_{z} \end{array}\right],$$

where $\zeta_{x}$, $\zeta_{y}$, $\zeta_{z}$ denote the filter compensation, according Equation (40), they can be defined

(53) $$\begin{array}{c} \left[\begin{array}{c} {\dot{\zeta }}_{x}\\ {\dot{\zeta }}_{y}\\ {\dot{\zeta }}_{z} \end{array}\right]=\left[\begin{array}{c} r\zeta_{y}-q\zeta_{z}\\ -r\zeta_{x}\\ q\zeta_{x} \end{array}\right]+\left[\begin{array}{c} -k_{x}{\tilde{x}}_{e}\\ -k_{y}\zeta_{y}\\ -k_{z}\zeta_{z} \end{array}\right]+\left[\begin{array}{c} {\dot{x}}_{ce}^{o}+{\dot{x}}_{ce}\\ {\dot{y}}_{ce}^{o}+{\dot{y}}_{ce}\\ {\dot{z}}_{ce}^{o}+{\dot{z}}_{ce} \end{array}\right]+\\ \left[\begin{array}{ccc} A & Bg(\tilde{\psi })u_{r} & Cg(\tilde{\theta })u_{r} \end{array}\right]\left[\begin{array}{c} \zeta_{u}\\ \zeta_{\psi }\\ \zeta_{\theta } \end{array}\right] \end{array},$$

where $\zeta_{\psi }$ and $\zeta_{\theta }$ are designed in section of stability analysis, $\zeta_{x}(0)=\zeta_{y}(0)=\zeta_{z}(0)=0$.

We choose the following Lyapunov function

(54) $$E_{1}=\frac{1}{2}(\sigma_{x}^{2}+\sigma_{y}^{2}+\sigma_{z}^{2}).$$

By differentiating Equation (54) along with Equations (53) and (40), we get

(55) $$\begin{array}{l} {\dot{E}}_{1}=-k_{x}\sigma_{x}^{2}-k_{y}\sigma_{y}^{2}-k_{z}\sigma_{z}^{2}\\ +\left[\begin{array}{ccc} \sigma_{x} & \sigma_{y} & \sigma_{z} \end{array}\right]\times \left[\begin{array}{ccc} A & Bg(\tilde{\psi })u_{r} & Cg(\tilde{\theta })u_{r} \end{array}\right]\left[\begin{array}{c} \zeta_{u}\\ \zeta_{\psi }\\ \zeta_{\theta } \end{array}\right]\\ =-k_{x}\sigma_{x}^{2}-k_{y}\sigma_{y}^{2}-k_{z}\sigma_{z}^{2}+A^{T}\left[\begin{array}{c} \sigma_{x}\\ \sigma_{y}\\ \sigma_{z} \end{array}\right]\sigma_{u}\\ +g^{T}(\tilde{\psi })B^{T}u_{r}\left[\begin{array}{c} \sigma_{x}\\ \sigma_{y}\\ \sigma_{z} \end{array}\right]\sigma_{\psi }+g^{T}(\tilde{\theta })C^{T}u_{r}\left[\begin{array}{c} \sigma_{x}\\ \sigma_{y}\\ \sigma_{z} \end{array}\right]\sigma_{\theta } \end{array}.$$

The filter compensation errors of the pitch and yaw angle can be defined as

(56) $$\begin{array}{l} \sigma_{\psi }=\tilde{\psi }-\zeta_{\psi }\\ \sigma_{\theta }=\tilde{\theta }-\zeta_{\theta } \end{array}.$$

According to Equation (44), $\zeta_{\psi }$ and $\zeta_{\theta }$ can be defined as

(57) $$\begin{array}{l} {\dot{\zeta }}_{\psi }=-k_{\psi }\zeta_{\psi }+\frac{(r_{ce}-r_{ce}^{o})+\zeta_{r}}{\cos\theta }\\ {\dot{\zeta }}_{\theta }=-k_{\theta }\zeta_{\theta }+(q_{ce}-q_{ce}^{o})+\zeta_{q} \end{array},$$

where $\zeta_{\psi }(0)=\zeta_{\theta }(0)=\zeta_{r}(0)=\zeta_{q}(0)=0$.

Choose the following Lyapunov function as

(58) $$E_{2}=\frac{1}{2}(\sigma_{\psi }^{2}+\sigma_{\theta }^{2}).$$

Differentiating Equation (58) alone with Equations (44) and (56), we get

(59) $$\begin{array}{l} {\dot{E}}_{2}=\sigma_{\psi }{\dot{\sigma }}_{\psi }+\sigma_{\theta }{\dot{\sigma }}_{\theta }=(\dot{\tilde{\psi }}-{\dot{\zeta }}_{\psi })\sigma_{\psi }+(\dot{\tilde{\theta }}-{\dot{\zeta }}_{\theta })\sigma_{\theta }\\ =(\frac{(r_{ce}-r_{ce}^{o})+\tilde{r}}{\cos\theta }-k_{\psi }\tilde{\psi }-\psi_{bs}+k_{\psi }\zeta_{\psi }-\frac{(r_{ce}-r_{ce}^{o})+\zeta_{r}}{\cos\theta })\sigma_{\psi }\\ +(-k_{\theta }\tilde{\theta }+(q_{ce}-q_{ce}^{o})+\tilde{q}-\theta_{bs}+k_{\theta }\zeta_{\theta }-(q_{ce}-q_{ce}^{o})-\zeta_{q})\sigma_{\theta }\\ =-k_{\psi }\sigma_{\psi }^{2}-k_{\theta }\sigma_{\theta }^{2}+\sigma_{q}\sigma_{\theta }-\theta_{bs}\sigma_{\theta }-\psi_{bs}\sigma_{\psi }+\frac{\sigma_{r}}{\cos\theta }\sigma_{\psi } \end{array},$$

where $\sigma_{q}=\tilde{q}$ and $\sigma_{r}=\tilde{r}$.

### 3.6. Design of Anti-Windup

The signal after the anti-windup scheme is present as

(60) $$u_{o}=\left\{ \begin{array}{l} u_{m},u\geq u_{m}\\ u,-u_{m}<u<u_{m}\\ -u_{m},u\leq -u_{m} \end{array}\right.,$$

where $u_{m}$ is the upper limited of control output. We design the anti-windup part as

(61) $$u=u_{o}-K_{o}\int (u-u_{o})dt,$$

where $K_{o}$ denote the anti-windup gain coefficient.

In summary, the main equations of the proposed path following controller are described as:

(62) $$\left\{ \begin{array}{l} F_{u}=(m-X_{\dot{u}})(-k_{u}\tilde{u}-k_{fu}{\mathrm{sig}}^{\gamma }(\tilde{u})+{\dot{u}}_{c}-u_{bs}-{\hat{F}}_{u})-f_{u}\\ M=(I_{y}-M_{\dot{q}})(-k_{q}\tilde{q}-k_{fq}{\mathrm{sig}}^{\gamma }(\tilde{q})+{\dot{q}}_{c}-q_{bs}-\hat{N})-f_{q}\\ N=(I_{z}-N_{\dot{r}})(-k_{r}\tilde{r}-k_{fr}{\mathrm{sig}}^{\gamma }(\tilde{q})+{\dot{r}}_{c}-r_{bs}-\hat{M})-f_{r} \end{array}\right.$$

**Figure 4:**
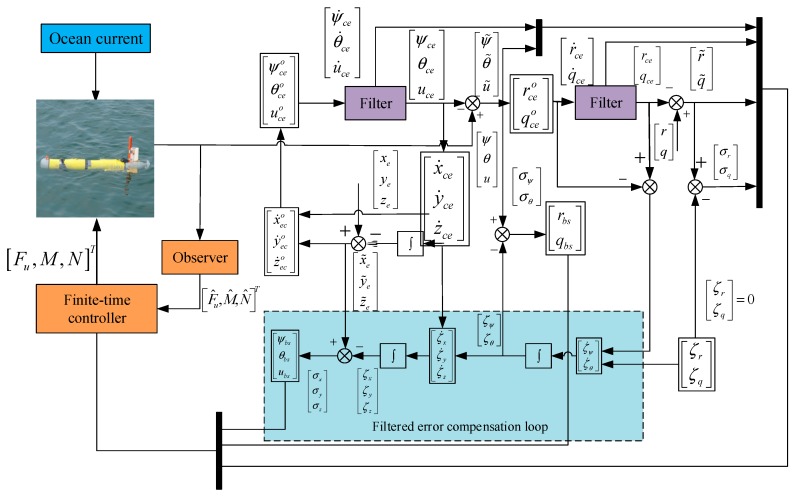
Structure of the present pat following control system.

Figure 4 shows the structure of the path following control system.

## 4. Stability Analysis

The stability of path following control system is proposed in this section. Firstly, we give the provident of the proposed controller without command filter. Substitute Equation (48) into Equation (7) and yield the following error equations

(63) $$\left\{ \begin{array}{l} {\dot{\sigma }}_{u}=-k_{u}\sigma_{u}-k_{fu}{\mathrm{sig}}^{\gamma }(\sigma_{u})-{\hat{F}}_{u}\\ {\dot{\sigma }}_{q}=-k_{q}\sigma_{q}-k_{fq}{\mathrm{sig}}^{\gamma }(\sigma_{q})-\hat{N}\\ {\dot{\sigma }}_{r}=-k_{r}\sigma_{r}-k_{fr}{\mathrm{sig}}^{\gamma }(\sigma_{r})-\hat{M} \end{array}\right..$$

Then Equation (64) can be present by

(64) $$\dot{\sigma }=-K_{p}\sigma -K_{f}sig^{\gamma }(\sigma )-\tilde{D},$$

where $K_{p}=diag(k_{u},k_{q},k_{r})$, $K_{f}=diag(k_{fu},k_{fq},k_{fr})$ and $\tilde{D}={\left[{\hat{F}}_{u},\hat{N},\hat{M}\right]}^{T}$.

A Lyapunov function candidate:

(65) $$V_{1}=\frac{1}{2}\sigma^{T}\sigma ,$$

where $\sigma ={\left[\sigma_{u},\sigma_{q},\sigma_{r}\right]}^{T}$ denote the attitude error, and differentiating Equation (66), we get

(66) $$\begin{array}{l} {\dot{V}}_{1}=\sigma^{T}\dot{\sigma }\\ =\sigma^{T}(-K_{p}\sigma -K_{f}sig^{\gamma }(\sigma )-\tilde{D})\\ =-\sigma^{T}K_{p}\sigma -\sigma^{T}K_{f}sig^{\gamma }(\sigma )-\sigma^{T}\tilde{D} \end{array},$$

where $\left|\tilde{D}\right|\leq \zeta_{M}$, according to Young’s inequality, it can be changed

(67) $$\sigma^{T}\tilde{D}\leq \left|\sigma^{T}\right|\zeta_{M}\leq \sigma^{T}\delta \sigma +\frac{1}{4}\zeta_{M}^{T}\delta^{-1}\zeta_{M},$$

where $\delta =diag(\delta_{1},\delta_{2},\delta_{3})$, $\delta_{i}$ are arbitrary positive constant.

Substitute Equation (68) into Equation (67), and we get

(68) $${\dot{V}}_{1}\leq -\sigma^{T}(K_{p}+\delta )\sigma -\sigma^{T}K_{f}sig^{\gamma }(\sigma )-\frac{1}{4}\zeta_{M}^{T}\delta^{-1}\zeta_{M}.$$

Furthermore, Equation (69) can be changed

(69) $$\begin{array}{l} {\dot{V}}_{1}\leq -\sigma^{T}(K_{p}+\delta )\sigma -\sigma^{T}K_{f}sig^{\gamma }(\sigma )\\ \leq -(K_{p}+\delta )V_{1}-2^{(\gamma +1)/2}K_{f}V_{1}^{(\gamma +1)/2} \end{array}.$$

According to the Lemma 2, the finite-time controller without command filter is finite-time stable. Additionally, convergence time can be expressed as follows: $t_{0}\leq \frac{1}{(K_{p}+\delta )\left(1-\gamma \right)/2}\ln\frac{(K_{p}+\delta )V_{1}^{\left(1-\gamma \right)/2}(t_{0})+2^{(\gamma +1)/2}K_{f}}{2^{(\gamma +1)/2}K_{f}}$.

Secondly, the stability of the overall control system is proposed. According to Equations (51), (54) and (58), we consider the following Lyapunov function

(70) $$V=E_{1}+E_{2}+\frac{1}{2}E^{T}KE,$$

where $K=diag\left\{k_{1},k_{2},k_{3}\right\}$ is a positive coefficient matrix. Let $G^{T}K+KG=-2Q$, and differentiating Equation (71), we get

(71) $$\begin{array}{l} \dot{V}={\dot{E}}_{1}+{\dot{E}}_{2}+E^{T}K\dot{E}\\ =-k_{x}\sigma_{x}^{2}-k_{y}\sigma_{y}^{2}-k_{z}\sigma_{z}^{2}-k_{\psi }\sigma_{\psi }^{2}-k_{\theta }\sigma_{\theta }^{2}-\frac{1}{2}E^{T}QE+A^{T}\left[\begin{array}{c} \sigma_{x}\\ \sigma_{y}\\ \sigma_{z} \end{array}\right]\sigma_{u}\\ +g^{T}(\tilde{\psi })B^{T}\left[\begin{array}{c} \sigma_{x}\\ \sigma_{y}\\ \sigma_{z} \end{array}\right]\sigma_{\psi }+g^{T}(\tilde{\theta })C^{T}\left[\begin{array}{c} \sigma_{x}\\ \sigma_{y}\\ \sigma_{z} \end{array}\right]\sigma_{\theta }+\frac{\sigma_{r}}{\cos\theta }\sigma_{\psi }+\sigma_{q}\sigma_{\theta }-\theta_{bs}\sigma_{\theta }-\psi_{bs}\sigma_{\psi }\\ -p_{21}\sigma_{u}u_{bs}-p_{22}\sigma_{q}q_{bs}-p_{23}\sigma_{r}r_{bs}+E^{T}KJsig^{\gamma }(E) \end{array}.$$

Then, to meet $\dot{V}<0$, the robust terms $\psi_{bs}$, $\theta_{bs}$, $u_{bs}$, $q_{bs}$ and $r_{bs}$ can be design as

(72) $$\begin{array}{l} \psi_{bs}=g^{T}(\tilde{\psi })B^{T}\left[\begin{array}{c} \sigma_{x}\\ \sigma_{y}\\ \sigma_{z} \end{array}\right],\theta_{bs}=g^{T}(\tilde{\theta })C^{T}\left[\begin{array}{c} \sigma_{x}\\ \sigma_{y}\\ \sigma_{z} \end{array}\right],u_{bs}=\frac{1}{p_{21}}A^{T}\left[\begin{array}{c} \sigma_{x}\\ \sigma_{y}\\ \sigma_{z} \end{array}\right]\\ q_{bs}=\frac{\sigma_{\theta }}{p_{22}},r_{bs}=\frac{\sigma_{\psi }}{p_{23}\cos\theta } \end{array}.$$

Substitute Equation (73) into Equation (72), and we get

(73) $$\begin{array}{l} \dot{V}={\dot{E}}_{1}+{\dot{E}}_{2}+E^{T}K\dot{E}\\ =-k_{x}\sigma_{x}^{2}-k_{y}\sigma_{y}^{2}-k_{z}\sigma_{z}^{2}-k_{\psi }\sigma_{\psi }^{2}-k_{\theta }\sigma_{\theta }^{2}-\frac{1}{2}E^{T}QE+E^{T}KJsig^{\gamma }(E) \end{array},$$

where J is a negative matrix. Hence, $\dot{V}<0$, it indicates that path following errors are convergent. The entire control close-loop system is global stable. The proof is completed.

## 5. Numerical Simulations

**Figure 5:**
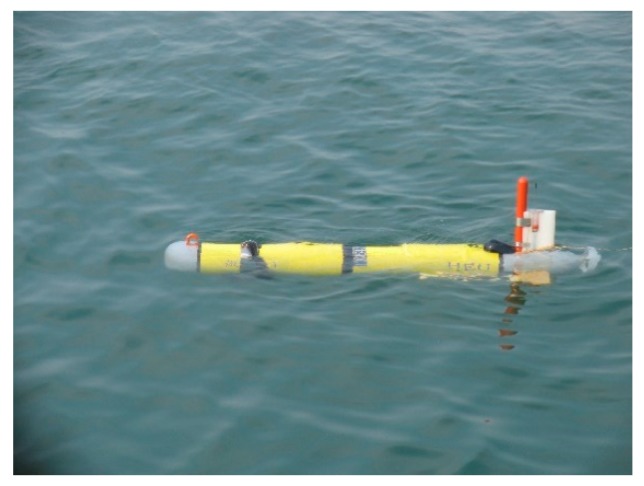
WL-4 underactuated AUV.

In this section, to verify and analyze the proposed controller (FTCFPC) base on the super-twisting observer, finite-time technology and command filtered backstepping methodology of this paper, numerical simulations on an underactuate AUV WL-4 (Figure 5)**.**, which is developed by Harbin Engineering University in China, were conduct in MATLAB-Simulink. 

In order to simulate a more realistic environment, the environment disturbances are considered in simulations. The disturbances can be expressed as

(74) $$d+T\dot{d}=K\varepsilon ,$$

where $d={\left[\tau_{ud},\tau_{vd},\tau_{wd},\tau_{qd},\tau_{rd}\right]}^{T}$ is the external disturbances vector. ε is a vector of white Gaussian noise with the largest amplitude of ε=1, and represents the high measurement noise. $K={\left[2,1,1,2,2\right]}^{T}$ represents the gain parameter matrix. $T={\left[20,20,20,20,20\right]}^{T}$ denotes the time constant matrix. 

For the underactuated AUV spiral dive operation, the following 3D desired path is design.

(75) $$\left\{ \begin{array}{l} x_{d}=20\cos(\frac{\pi }{10}s)\\ y_{d}=20\sin(\frac{\pi }{10}s)\\ z_{d}=s \end{array}\right..$$

The initial position of the AUV is $(x_{0},y_{0},z_{0})=(30,0,1)m$, the initial attitude angle are $\left(\theta_{0},\psi_{0})=(0,0\right)$. The initial velocities of AUV are $v_{0}=0m/s$, $u_{0}=0m/s$, and $w_{0}=0m/s$. The desired velocity is $u_{d}=1m/s$. The constant velocity of ocean currents is v=(0.2,0.2,0.05)m/s. The path following control simulations results using FTCFPC control are compared with the results of traditional backstepping.

In the numerical simulation, we design AUV controllers according to (47) and the parameters are given by $k_{x}=5$, $k_{y}=1$, $k_{z}=2$, $k_{\psi }=k_{\theta }=2$, $k_{u}=8$, $k_{q}=k_{r}=3$, $k_{fu}=k_{fq}=k_{fr}=1$, $p_{21}=5$, $p_{22}=p_{23}=5$. The parameters of the filter are selected as $w_{n}=20rad/s$, ζ=0.9.

**Figure 6:**
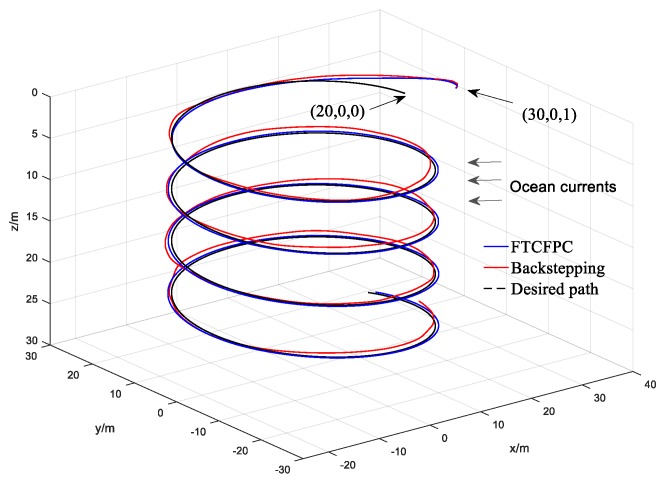
AUV following the 3D path.

**Figure 7:**
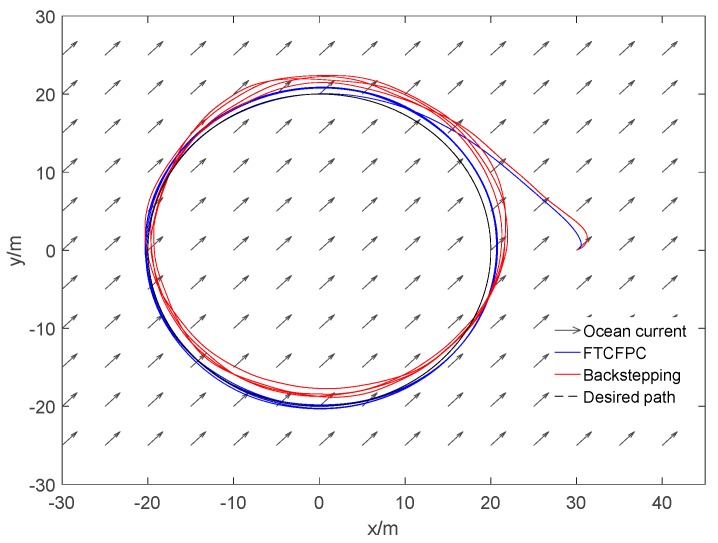
Resultant curves of horizontal projection.

**Figure 8:**
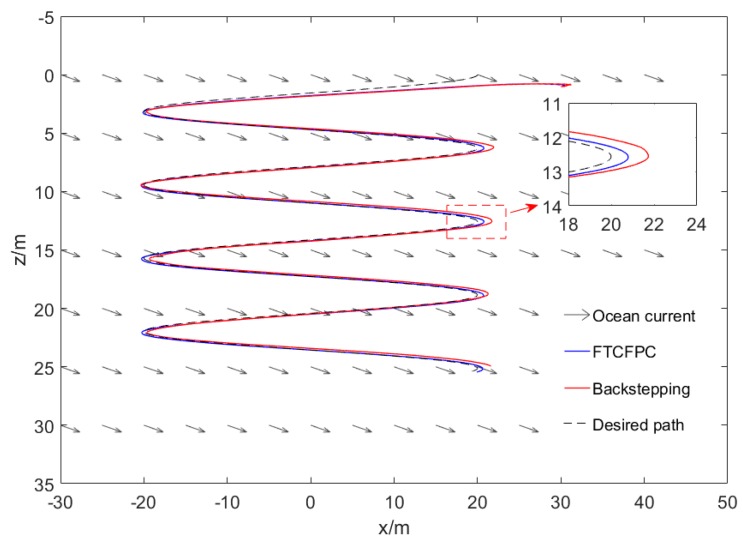
Resultant curves of vertical projection.

**Figure 9:**
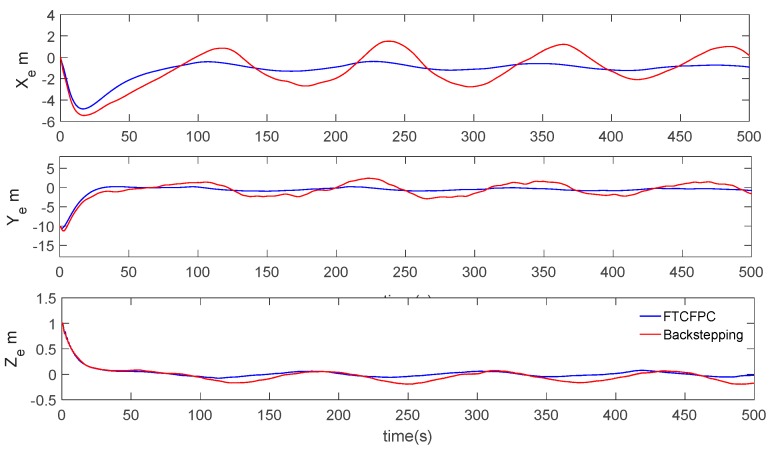
Resultant curves of path following errors.

**Figure 10:**
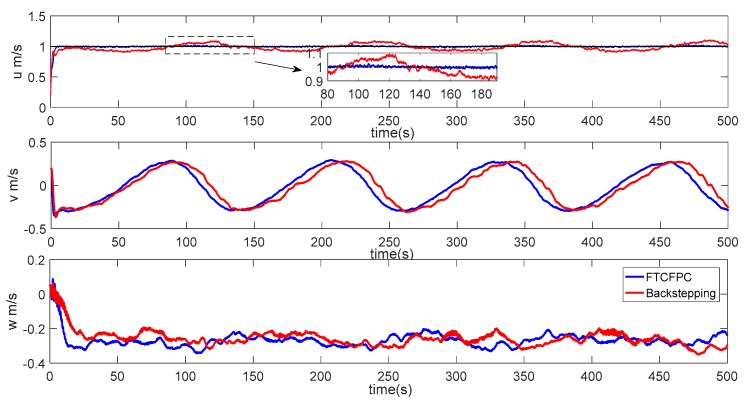
Velocity response of AUV.

**Figure 11:**
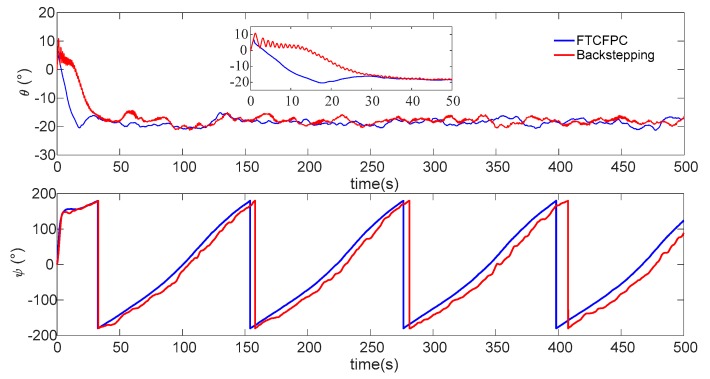
Angle response of AUV.

**Figure 12:**
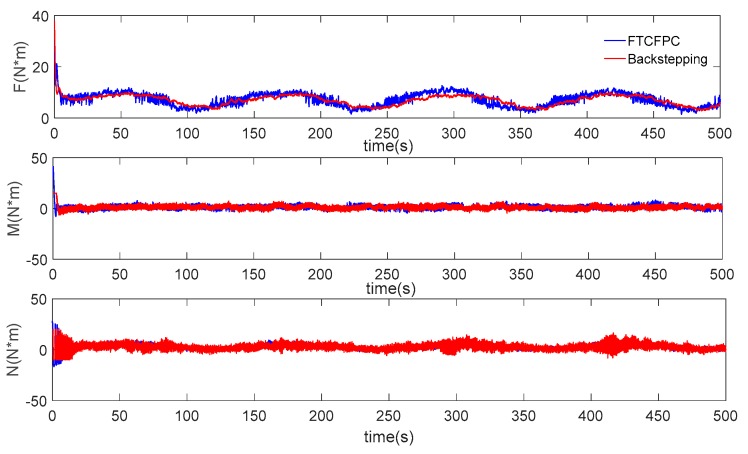
Control force and moments of AUV.

Finally, the simulation results by using finite-time command filtered backstepping control and the traditional backstepping control are shown in Figure 6, Figure 7, Figure 8, Figure 9, Figure 10, Figure 11 and Figure 12.

The real and desired paths in the spatial space are shown in Figure 6, Figure 7 and Figure 8 respectively show the projection curves of horizontal plane and vertical plane. It can be seen from these figures that the actual path of the AUV deviates from the desired path due to the influence of the ocean current. As can be seen from Figure 6, Figure 7 and Figure 8. The path under FTCFPC control is closer to the desired path than that under traditional backstepping control. The FTCFPC controllers has the smaller offset and achieve significantly higher accuracy than traditional backstepping controllers in the presence of unknown disturbance and ocean currents. Figure 9 shows the tracking errors of 3D path. It can be seen that the error of real and desired path with the FTCFPC controller can converge in a short time. The position error of two controllers has some fluctuations, but the fluctuation of the FTCFPC method is much smaller. These can be proved that the FTCFPC method present in this paper has good robustness and effectiveness. Figure 10 and Figure 11 depict the response of velocity and angle respectively, from which we can see that, FTCFPC method can achieve the desired path more quickly and has good anti-interference ability. The control signals of different channels are shown Figure 12.

In summary, the FTCFPC method proposed in this paper can make the AUV tracking to the desired path smoothly and robustly with the ocean currents. Following are the advantages which can been seen from the above results:

1. The controller has strong robustness under the interference of ocean current.
2. It has faster convergence, and the AUV can follow the desired path in a short time.
3. The control has better following accuracy under current disturbance.

## 6. Conclusions

This paper proposes a FTCFPC controller to improve an underactuated AUV path following performance in the presence of ocean currents and unknown disturbances. The designed controller is based on command filtered backstepping method, finite-time theory and super-twisting observer techniques. A second-order filter is designed to achieve derivative of virtual control signals such that the computational complexity can be reduced and the differential expansion can be avoided. A filtered error compensation loop is developed to ensure the error between the desired signals and the filter signals converge. A super-twisting observer is proposed to reduce the impact of unknown disturbances and ocean currents. A finite-time controller is introduced to ensure the system can be stable in a short time. The system stability is analyzed based on the Lyapunov stability theory. Simulation results indicates that the 3D path following controller proposed in this paper for underactuated AUVs is more effective and robust than the conventional backstepping method in the presence of ocean currents and unknown disturbances.

For the future works, more real external disturbance will be considered such as impermanent currents and waves near the surface. The cooperative path following of AUVs and the limited performance of actuators should be developed.
